# Portable six-channel laser speckle system for simultaneous measurement of cerebral blood flow and volume with potential applications in characterization of brain injury

**DOI:** 10.1117/1.NPh.12.1.015003

**Published:** 2025-01-24

**Authors:** Simon Mahler, Yu Xi Huang, Max Ismagilov, David Álvarez-Chou, Aidin Abedi, J. Michael Tyszka, Yu Tung Lo, Jonathan Russin, Richard L. Pantera, Charles Liu, Changhuei Yang

**Affiliations:** a California Institute of Technology, Department of Electrical Engineering, Pasadena, California, United States; b University of Southern California, USC Neurorestoration Center and Department of Neurological Surgery, Los Angeles, California, United States; c California Institute of Technology, Division of Humanities and Social Sciences, Pasadena, California, United States; d Rancho Los Amigos National Rehabilitation Center, Downey, California, United States; e Kaweah Health Medical Center, Neurology, Visalia, California, United States

**Keywords:** brain injury, speckle contrast optical spectroscopy, cerebral blood flow, laser speckle contrast imaging, biomedical optics, non-invasive brain imaging

## Abstract

**Significance:**

Cerebral blood flow (CBF) and cerebral blood volume (CBV) are key metrics for regional cerebrovascular monitoring. Simultaneous, non-invasive measurement of CBF and CBV at different brain locations would advance cerebrovascular monitoring and pave the way for brain injury detection as current brain injury diagnostic methods are often constrained by high costs, limited sensitivity, and reliance on subjective symptom reporting.

**Aim:**

We aim to develop a multi-channel non-invasive optical system for measuring CBF and CBV at different regions of the brain simultaneously with a cost-effective, reliable, and scalable system capable of detecting potential differences in CBF and CBV across different regions of the brain.

**Approach:**

The system is based on speckle contrast optical spectroscopy and consists of laser diodes and board cameras, which have been both tested and investigated for safe use on the human head. Apart from the universal serial bus connection for the camera, the entire system, including its battery power source, is integrated into a wearable headband and is powered by 9-V batteries.

**Results:**

The temporal dynamics of both CBF and CBV in a cohort of five healthy subjects were synchronized and exhibited similar cardiac period waveforms across all six channels. The potential use of our six-channel system for detecting the physiological sequelae of brain injury was explored in two subjects, one with moderate and one with significant structural brain damage, where the six-point CBF and CBV measurements were referenced to structural magnetic resonance imaging (MRI) scans.

**Conclusions:**

We pave the way for a viable multi-point optical instrument for measuring CBF and CBV. Its cost-effectiveness allows for baseline metrics to be established prior to injury in populations at risk for brain injury.

## 1 Introduction

Brain injury can occur from traumatic and non-traumatic mechanisms. Traumatic brain injury (TBI) is one of the leading causes of death and disability among young people worldwide.^1^^–^^3^ A TBI occurs when the brain experiences excessive non-physiological mechanical forces, which can lead to hemorrhage, contusion, inflammation, cell death, edema, and/or ischemia. After recovery from TBI, patients can exhibit persistent evidence of structural brain damage as seen on magnetic resonance imaging (MRI), as well as physiological disruptions such as cerebrovascular dysregulation that lead to subtle functional deficits that may worsen with time if left untreated.^4^^,^^5^ Although structural sequela of TBI can be readily characterized by MRI, objective measures of cerebrovascular reactivity and cerebral blood flow (CBF) can be very helpful in fully characterizing the effect of TBI,^3^ including mild cases not associated with obvious structural damage to the brain. The incidence of mild TBI with minimal structural brain damage is particularly high—there are nearly three million mild TBI occurrences in the US each year with the majority occurring in adolescents and young adults. Mild TBI is also one of the leading causes of injuries in the U.S. Army, with blast-related TBI often described as the signature injury during deployment.^6^^–^^8^ Despite the heavy injury toll and almost two decades of research, the diagnosis, treatment, and recovery from TBI remain poorly understood. Various methods have been studied for characterizing TBI, including MRI-based neuroimaging,^9^ electrophysiology,^3^^,^^10^ and blood and saliva biomarkers.^11^^–^^13^ Beyond TBI, non-traumatic brain injury (NTBI) can also lead to structural and physiological sequela, including conditions such as stroke, hypoxia, infections, and toxic exposures. NTBI often results from vascular events or progressive neurodegenerative disorders, which disrupt blood flow to critical brain regions, causing localized or widespread neuronal damage. In addition, conditions such as hypoxic-ischemic injury or encephalitis can impair cerebrovascular function and lead to long-term cognitive, motor, and sensory deficits. These injuries can result in damage to brain tissue and persistent disruption of normal cerebrovascular reactivity, emphasizing the need for cost-effective, comprehensive, and reliable methods that extend beyond structural imaging to assess functional impairments in cerebrovascular dynamics and CBF.^14^

**Fig. 1:**
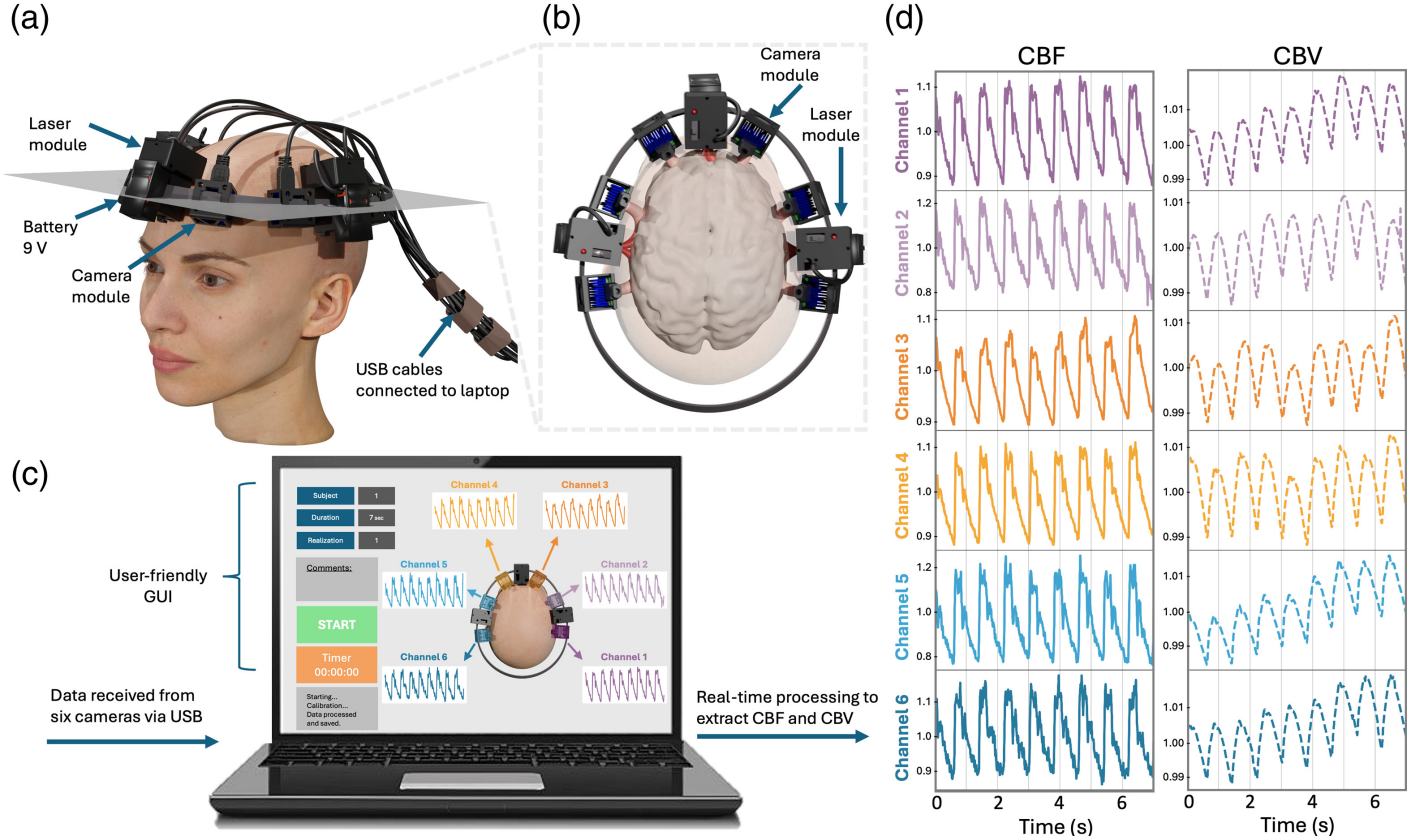
Experimental arrangement of the six-channel SCOS system. (a) 3D visualization of the six-channel system positioned on the head. (b) Schematic of the light penetrating the brain. (c) GUI for operating the system and for real-time visualization of the CBF and CBV. (d) Typical CBF and CBV blood dynamics measured at six different locations of the head from our compact SCOS system.

Recently, laser speckle contrast imaging was applied for monitoring CBF and cerebral blood volume (CBV) non-invasively in humans.^15^^–^^20^ The technique, commonly called speckle contrast optical spectroscopy (SCOS)^16^^–^^21^ or speckle visibility spectroscopy,^15^^,^^21^^–^^25^ is an offshoot of laser speckle contrast imaging and uses an infrared laser source to transmit light through the skull and brain in humans [Fig. 1(a)}. By transmitting infrared light through one location on the skull and collecting its transmission with a camera in another location, it is possible to determine brain blood volume by measuring the light attenuation rate.^17^^,^^21^ As the light used is also coherent (laser), it is possible to determine the brain blood flow rate by recording how fast the transmitted speckles fluctuate.

In this paper, we present a compact and portable six-channel SCOS system capable of simultaneously measuring CBF and CBV at six different brain locations (Fig. 1). The system includes three laser diode modules and six universal serial bus (USB) board camera sensor modules, which can be conveniently positioned around the subject’s head at distinct locations. Apart from the camera cables’ connection to a laptop, the entire system is integrated into a wearable headband and is powered by embedded and rechargeable 9-V batteries. Data are transmitted via USB for real-time processing, enabling operation in diverse environments. The camera modules are designed to dissipate heat effectively, ensuring a comfortable contact temperature during extended recordings. In addition, a graphical user interface (GUI) enables non-experts to operate the portable six-channel SCOS system with ease. We characterized and tested the system on a cohort of five healthy human subjects. The results showed synchronized and highly correlated dynamics of blood flow and blood volume across all six channels. Comfort evaluation tests confirmed the wearable system’s comfort during recording and established the system’s maximum operational duration.

Compared with existing SCOS systems such as those developed in Refs. 17–19, our proposed multi-channel, compact SCOS system offers some differences. First, it utilizes head-mounted, battery-powered continuous-wave laser diodes instead of pulsed lasers. This eliminates the need for pulsing electronics and reduces both the system’s cost and size. Second, the modular design of our setup—with isolated sources and detectors—provides greater operator flexibility. Last, by directly mounting the camera a few millimeters above the skin with no aperture between the camera and the skin, we maximize photon capture and improve the signal-to-noise ratio. Further details on these improvements can be found in Ref. 16. Due to these, our multi-channel SCOS system can be mounted around the head.

As a preliminary investigation, we explored the potential use of our six-channel system for detecting the persistent effects of brain injury, whether traumatic or non-traumatic, using time-synchronized measurements of blood flow and blood volume at different brain locations, which offer a physiology-based approach to assess regional CBF and CBV differences, with suspected injured regions displaying different blood dynamics. We conducted a preliminary study involving two subjects who have a history of brain injury, one traumatic and the other non-traumatic. Each of the two subjects has undergone decompressive hemicraniectomy and cranioplasty but with different degrees of persistent structural damage to the brain, and we compared their results with those of the five healthy subjects. For the subject with significant structural brain damage, the six-point CBF and CBV measurements were compared with MRI scans to correlate the location of structural damage and altered blood dynamics.

## 2 Methods

### 2.1 Six-Channel SCOS System

The arrangement of our compact six-channel SCOS system, shown in Fig. 1, builds upon the single-channel SCOS device reported in Ref. 16. The wearable headband and location of each channel on the head are shown in Fig. 1(a). The system contains three laser sources for illumination and six board cameras for detection. The cameras are distributed on the head as follows: two on each side of the forehead: one on the front and one on the back of the left hemisphere, and one on the front and one on the back of the right hemisphere. Each camera is placed at a source-to-detector (S-D) distance from the laser source around 3.2±0.2  cm [see Fig. 1(b)]. It was previously reported that an S-D distance between 3.0 and 3.5 cm corresponds to an optimal brain sensitivity over signal-to-noise ratio.^15^^,^^21^^,^^26^^–^^30^ We note that, even at such a large S-D distance, the collected signal would still be influenced by blood flow in the scalp.

**Fig. 2:**
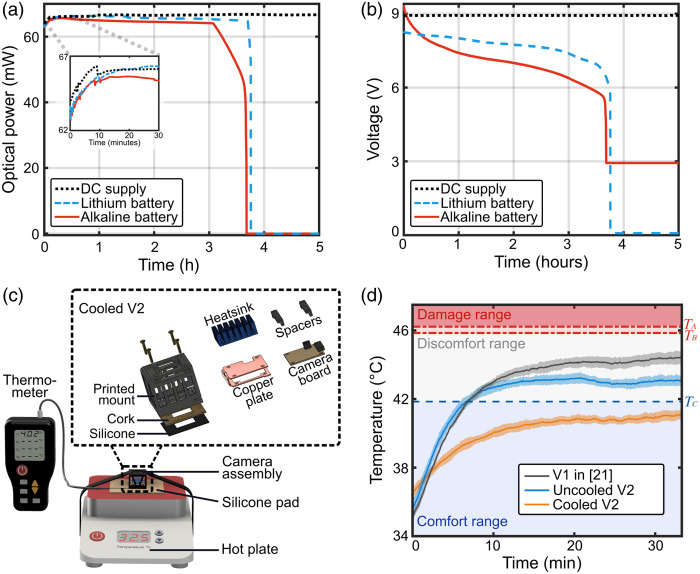
Optical lasing power and camera heating temperature characterization of the system. (a) Optical power and (b) power supply voltage plotted as functions of laser operating time. (c) Diagram of the camera module, including an exploded view of the heat management system (inset). (d) Contact temperature measured as a function of the camera’s operating time.

Each laser source is a single-mode continuous wave laser diode of 808 nm (Thorlabs M9-808-0150, Newton, New Jersey, United States). To ensure control over the illumination spot size and prevent undesirable laser light reflections or stray light, the laser diode is housed in a 3D-printed mount. A sliding block switch at the end of the laser mount can block laser light when the system is not in use. The camera is also housed in a 3D-printed mount. The mounts were printed using the Anycubic Photon Mono X 6K, a resin-based [stereolithography (SLA)/digital light processing (DLP)] printer, with black Anycubic Water-Wash + resin. The use of black resin was intentional to optimize light absorption and reduce back reflection and stray light. After printing, the components were fully cured using UV light to ensure safe handling. To further ensure biocompatibility and sanitization, a 1-mm silicone sheet was applied to the 3D-printed parts, ensuring that the device meets hygiene standards and is suitable for direct skin contact in clinical settings. The total illumination power is limited to 67 mW to ensure that the laser light intensity level of the area of illumination is well within the American National Standards Institute (ANSI) laser safety standards for maximum permissible exposure ($3.28\;\mathrm{mW}/{\mathrm{mm}}^{2}$) for skin exposure to an 808-nm laser beam.^31^ For that, the laser diode is positioned 6 mm away from the skin of participants such that the illumination spot diameter was 5.5 mm, as successfully used in Ref. 16. This spot size prevents excessive power density, balances safety and performance, and ensures adequate S-D distance resolution for separations greater than 3 cm. To limit the output power to 67 mW, each laser is operated by a custom-made printed circuit board combined with a laser diode driver (Thorlabs LD1100), both encased atop the laser diode [see Fig. 1(a)]. The laser power is adjusted by tuning the onboard potentiometer on the compact laser driver board (Thorlabs LD1100). On the back of the laser mount, a 9-V battery provides electrical energy for switching the three laser sources with battery life lasting >3  h of usage [Figs. 2(a) and 2(b)].

The camera used is a rolling shutter camera [Basler daA3840-45um (Sony IMX334 sensor), Ahrensburg, Germany] operating at a framerate of 40 frame-per-second, with a pixel pitch of 2×2  μm, a pixel resolution of 3840×2160  pixels, and a pixel depth of 8 bits, operated at an exposure time T=6  ms. See the Supplementary Material for more details about the choice of this camera over the Basler daA1920-160um camera used in Ref. 16.

The design of the camera module is illustrated in Fig. 2(c). A heat management system is incorporated to ensure that the contact temperature of the camera module remains below the safety threshold. This passive system [Fig. 2(c)] is composed of multiple layers, starting with a 1-mm-thick black silicone rubber sheet in contact with the skin, followed by a 1-mm-thick cork sheet and a 3D-printed encasing mount. Inside the mount, a 1-mm (0.040″) thick laser-cut copper plate, bent into a U shape, encases a Basler daA3840-45um board camera. On the back of the copper plate, an anodized aluminum heat sink measuring 30×20×10  mm is attached to dissipate heat effectively. The silicone and cork sheets are precision-cut and adhered to the front face of the 3D-printed mount. The camera board is securely housed within the U-shaped copper plate and 3D printed mount using screws and bolts with spacers. To prevent electrical contact, insulating electrical tape is applied between the camera and the edges of the copper plate. The heat sink is affixed to the back of the copper plate using thermal paste for enhanced heat transfer, along with a thin layer of glue at each corner to ensure stability.

The cameras are connected to a laptop via USB 3.0 cables. To avoid potential conflicts arising from using USB hubs, we used a laptop with six independent USB ports (Lenovo Legion 5 Pro). Each port operates independently, ensuring stable data transfer from all six cameras. The IMX334 sensor can operate up to a sampling rate of 45 frames per second (FPS), which saturates the 5 Gbps USB 3.0 bandwidth per port. To mitigate this, we capped the sampling rate at 40 FPS. Scaling the system beyond six channels would require alternative solutions such as Thunderbolt-to-PCIe docks or PCIe-to-USB cards. The total hardware cost of the six-channel SCOS system is ∼$3000, with data collection and processing carried out on the Lenovo Legion Pro laptop equipped with an Nvidia GeForce RTX 4070 GPU, priced around $1500.

### 2.2 CBF and CBV Measurements

In this section, we briefly describe the methods for extracting CBF and CBV from speckle camera images. A detailed explanation of the methodology is available in Ref. 16, where we introduced a compact, single-channel SCOS device for measuring CBF with high sensitivity and temporal resolution at large S-D distances. The method for extracting CBV was detailed in Ref. 21.

The estimated number of speckles per pixel collected by the camera is about four speckles per pixel, corresponding to a one-dimensional speckle-to-pixel length ratio of s/p=0.5.^24^^,^^32^^,^^33^ As demonstrated previously, fully resolving individual speckles is not required to extract dynamic information in SCOS systems.^16^^,^^24^^,^^32^^,^^33^ Optimal signal-to-noise ratio can be achieved when the s/p ratio is <1, as discussed in Refs. 24, 32, and 33, as well as in the Supplementary Material of our previous work on compact SCOS.^16^

The speckle contrast K is calculated from the recorded speckle images as

$$K_{\text{raw}}^{2}(I(t))=\frac{\sigma^{2}(I(t))}{\mu^{2}(I(t))}.$$ (1)

where σ(I(t)) is the standard deviation of the normalized image I at time t is and μ(I(t)) its mean. The various sources of noise accounted as^16^^,^^18^^,^^34^^,^^35^

$$K_{\text{adjusted}}^{2}(t)=K_{\text{raw}}^{2}(t)-K_{\text{shot}}^{2}(t)-K_{\text{quant}}^{2}(t)-K_{\mathrm{cam}}^{2}(t),$$ (2)

with $K_{\text{shot}}^{2}$ accounting for variance contributions from the shot noise, $K_{\text{quant}}^{2}$ for the variance inherited from quantization, and $K_{\mathrm{cam}}^{2}$ for the variance contributions of the camera’s readout noise and dark noise. See Refs. 16 and 18 for more details about the speckle contrast calculations and calibration processes. The cerebral blood flow index (CBFI) is related to $K_{\text{adjusted}}^{2}$ by

$$\mathrm{CBFI}(t)=\frac{1}{K_{\text{adjusted}}^{2}(t)}.$$ (3)

The blood volume is extracted from the camera images by calculating the cerebral blood volume index (CBVI) from the recorded images as^17^^,^^21^

$$\mathrm{CBVI}(t)=\frac{2I_{0}-\mu (I(t))}{I_{0}},$$ (5)

where $I_{0}$ is the intensity at baseline, calculated as the mean of the average intensity of the entire image acquired during the baseline period. The mean μ(I(t)) is calculated as the average spatial intensity of the entire image I acquired at time t and can be expressed in the camera grayscale unit. In the results of this paper, we utilize normalized CBFI and CBVI metrics to provide normalized blood dynamics information for enhanced comparability across measurements. For better consistency with previous works of compact SCOS in Refs. 17 and 21, we used in Eq. (5) the linear approximation of the CBV formula. Although this approximation works well for blood volume measurement, in retrospect, we note that particularly for scenarios involving acute or significant blood volume changes, the CBV formula can be improved using the logarithmic definition, as defined in Ref. 18,

$$\Delta \mathrm{CBVI}(t)=\log_{10}(\frac{I_{0}}{\mu (I(t))}).$$ (6)

### 2.3 Graphical User Interface

A GUI, illustrated in Fig. 1(c), controls the system and displays the CBF and CBV results at the end of each recording. The Python-based GUI prompts users to input details such as subject information, trial number, and the duration of the recording. The GUI caps the recording duration to 180 s. Users can also add comments regarding the qualities of the recording, potential movements, and subject information. Furthermore, the GUI ensures safety by requiring users to confirm adherence to all laser safety protocols before initiating measurements. Upon completion, the GUI immediately presents the results, including CBF and CBV for each channel [Fig. 1(c)]. The real-time processing of data negates the need to save raw images, thereby optimizing disk space usage. We plan to incorporate a live-view mode into the GUI using a sliding window approach similar to that in Refs. 25 and 36, enabling real-time visualization of CBF and CBV.

Figure 1(d) shows typical CBF and CBV time traces measured over 5 s by the six-channel SCOS system on a human subject. As shown, all CBF and CBV time traces exhibit the same frequency of oscillation and are temporally synchronized. Note that the CBF time traces contain more high-frequency information than the CBV time traces, including variations such as the peak systole or dicrotic notch of the cardiac pulse.^17^^,^^18^^,^^21^^,^^37^^,^^38^ In addition, the CBF time traces show greater temporal stability compared with CBV.

### 2.4 Battery-Powered Laser Source and Heat Management in Camera Modules

Next, we evaluated the stability and longevity of the laser source when powered by a direct current (DC) power supply versus a 9-V battery [Figs. 2(a) and 2(b)] and examined the contact temperature of the camera module [Figs. 2(c) and 2(d)]. These characterizations are critical for using the system in a clinical setting as patients in such settings may not be able to respond to pain or discomfort, or they may be unconscious. Therefore, it is essential to first ensure that powering the system with a low-voltage battery guarantees electrical safety for the patients being scanned and that the system’s temperature is carefully constrained to prevent any risk of heat-induced damage.

Figure 2(a) shows the laser output power, measured with an optical power meter positioned on the mounted laser diode, for different power sources: a 9-V DC bench power supply, an alkaline battery (Amazon Basics 6LR61), and a rechargeable lithium-ion battery (EBL^®^ 6F22, 600 mAh at 3.7V, 2 cells). We also tested a zinc chloride battery (Thunderbolt Magnum 9 V, Fullerton, California, United States), but it lasted <5  min, so we decided not to include it in the results.

As shown, all power sources provided the laser with an optical power of ∼66  mW over time. The DC power supply maintained a constant output, whereas the alkaline and rechargeable lithium-ion batteries’ power decreased to zero after a few hours due to discharge. Overall, the lithium-ion battery outperformed the alkaline battery, lasting the longest (∼3.7  h), demonstrating the highest stability in optical power output, and being the most lightweight. Detailed performance comparisons of the batteries can be found in the Supplementary Material. In addition, the system optical power with the rechargeable lithium-ion battery sharply dropped to 0 within 3 min due to an internal electrical switch that automatically disables the battery when the voltage falls below a certain threshold. In our case, this feature is beneficial as it prevents noisy data collection when the battery is depleted. With an average measurement time of 15 min per subject, the lithium battery can support up to 12 recordings or more on a single charge. This is practical as the battery can be recharged between sessions.

Next, we tested the effectiveness of a passive heat management system for the camera modules by measuring the contact temperature after placing the system on silicone skin pads (Stylia) positioned on a hot plate (brand VWR) set at 32.5°C to simulate human skin contact [Fig. 2(c)]. Our goal was to ensure compliance with thermal guidelines for skin contact (ASTM C1055^39^ and IEC60601,^40^ West Conshohocken, Pennsylvania, United States) and to provide user comfort during extended system operation. From the ASTM guidelines, we established three temperature thresholds for 1 h of operation of the system: (1) a comfort temperature threshold $T_{A}=41.85{}^{\circ}C$, (2) a reversible skin damage threshold temperature $T_{B}=45.85{}^{\circ}C$, and (3) a severe skin damage threshold temperature $T_{C}=46.22{}^{\circ}C$. Our aim was to not overcome the comfort temperature $T_{A}$. We tested three camera modules. The first (V1) was the camera module (Basler daA1920-160um) used in Refs. 16 and 21 running at 80 FPS with 2.3 million pixels, and the second (uncooled V2) was a Basler daA3840-45um board camera running at 30 FPS and 8.3 million pixels. The third (cooled V2) was the camera module we used in the rest of this paper. It consists of a camera module with a Basler daA3840-45um board camera running at 40 FPS and 8.3 million pixels encased in the heat management system shown in Fig. 2(c). For heat management, we attached an anodized aluminum heatsink to a C-shaped copper plate containing the camera board. On the front side, we incorporated a 1-mm layer of cork and a 1-mm layer of neoprene to further insulate heat. For experiments involving human subjects, we replaced the neoprene sheet with a silicone sheet to allow for easier and more thorough sanitization. The average temperature results for each module are presented in Fig. 2(d), where the camera was continuously run for a period of 35 min (i.e., a duty cycle of 100%). As shown, all the cameras’ temperatures remained below both the reversible $T_{B}$ and irreversible $T_{C}$ damage thresholds, in accordance with ASTM guidelines. However, only the cooled V2 module stayed within the targeted comfort temperature range $T_{A}$.

The camera resolution (3840×2160) in the current setup [cooled V2]^16^^,^^21^ limits the frame rate to 45 FPS due to USB 3.0 bandwidth constraints. To ensure stability, the cooled V2 system operates at 40 FPS. The uncooled version is limited to 30 FPS due to thermal throttling at higher frame rates as it cannot complete a 35-min stress test without dropping frames. However, this comparison still showcases our cooling system’s efficiency as the heat management system could lower the camera temperature by at least 3°C while enabling a higher frame rate of 40 FPS without throttling or instability.

### 2.5 Human Research Study

All human subjects research was performed with informed consent under a protocol (IR21-1074) approved by the Caltech Institutional Review Board. Participants were recruited from a pool of adults aged 18 to 70 years in the greater Los Angeles area. Before the experiments, each participant completed a health history questionnaire, and their blood pressure was recorded. To simplify the experimental setup and avoid scattering from scalp hair, measurements were taken on areas with minimal or no hair. The total illumination power adhered to the ANSI laser safety standards for maximum skin exposure to an 808-nm laser beam.^31^

A total of seven subjects were enrolled in the study: five healthy subjects with no history of brain injury and two subjects with a history of brain damage. One subject suffered TBI with a cranioplasty skull implant but no major structural brain damage at the time of the study. The second subject suffered NTBI from a large brain hemorrhage due to a ruptured arteriovenous malformation (NTBI) and undergoing a cranioplasty skull implant procedure at the time of the study. Both subjects underwent decompressive hemicraniectomy and subsequent cranioplasty and retained a skull implant. The NTBI patient had more evidence of structural brain damage and was still in post-discharge care due to behavioral issues at the time of the study. The TBI patient had relatively less structural brain damage and was living independently. Neither had focal neurological deficits.

To simplify the experiment and system implementation, measurements were conducted on hairless areas or regions with minimal hair, no more than a few millimeters long. For that, all subjects had their head shaved. The optical transmission of our system is optimal when both the light source and detector are positioned in areas with little to no hair, ideally, a hair-free square area of $1.5\;{\mathrm{cm}}^{2}$. Although these hair-free areas are easily attainable for subjects with short hair, they are not for participants with longer hair. To address long hair interfering with system use, we plan on designing 3D-printed laser and camera mounts equipped with hair separators in the area of interest. This addition would minimize hair interference with the optical transmission process and would enable the system to potentially be used on participants with longer hair.

## 3 Results

As shown in Fig. 1(d), our multi-channel SCOS system can simultaneously measure both CBF and CBV dynamics at six distinct locations on the head. For physiological monitoring systems, characterizing the system’s contact temperature during operation is critical to ensuring participant safety. This is particularly important for systems in use in clinical settings, where patients may be unable to respond to pain or discomfort or may be unconscious.^41^ Although the laser source of our system adheres to ANSI standards, limiting the optical power level to prevent heat induction in participants, the detection module, working at a high speed, generally generates heat.^42^^,^^43^ In systems where the modules are in direct contact with the participants’ skin, heat becomes a significant concern,^41^^–^^43^ especially as these units typically operate at high speeds. To mitigate this issue, detecting devices can be operated for only a short period of time, as short as a few minutes, or are switched on and off to allow for cooling between scans. Although these methods provide partial solutions, they are not practical for non-expert users and still pose safety risks. To address these challenges, we have designed a passive heat modulation system, as illustrated in Fig. 2, enabling our system to operate for more than half an hour while maintaining a comfortable temperature range. Passive cooling offers the advantage of regulating temperature without the need for complex or vibrating equipment with active external energy sources, making it more user-friendly and more practical for long-term use.^43^^,^^44^

**Fig. 3:**
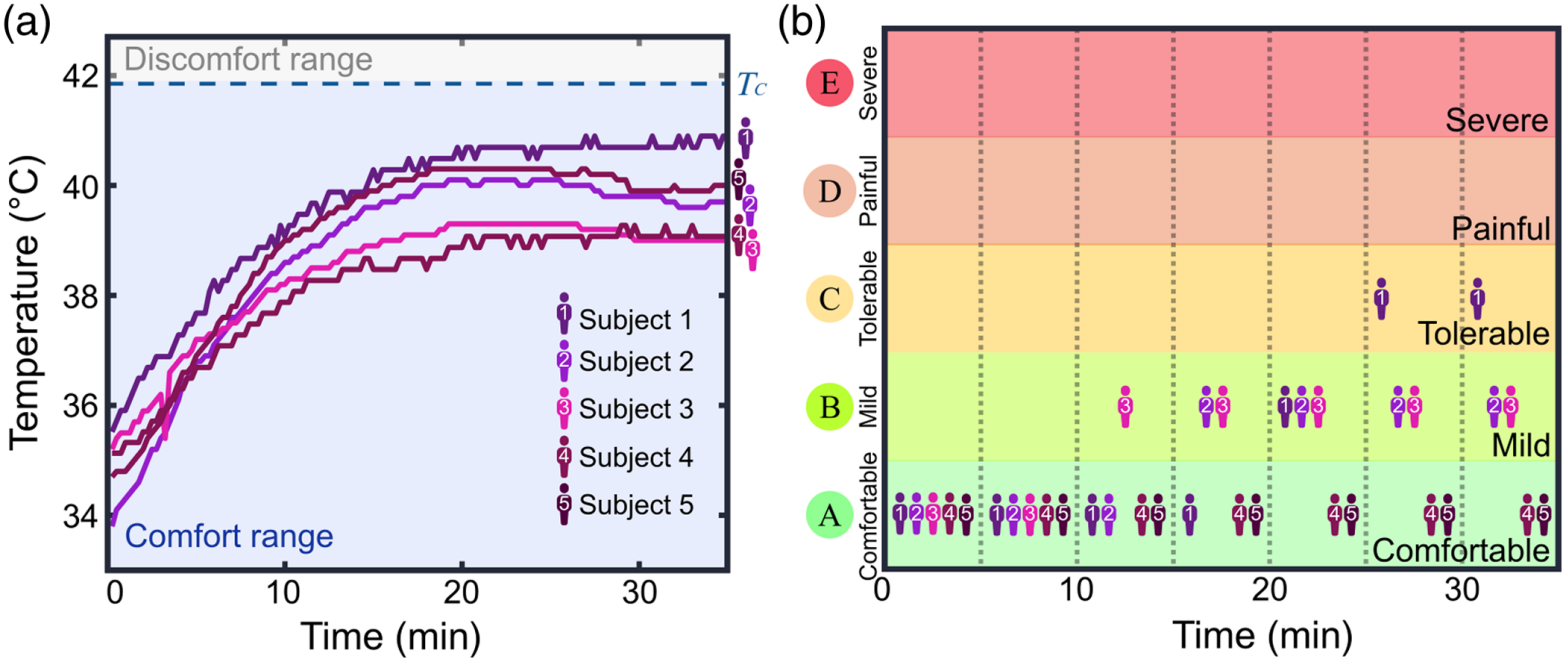
(a) Temperature measurements taken at the contact surface of the camera module onto the foreheads of five human subjects. The measured temperature saturated after 20 min of continuous operation and always remained within the comfort range (i.e., below $T_{A}=41.85{}^{\circ}C$). (b) Comfort ratings provided by participants during the 35 min of recording. The room temperature was 23°C.

In Fig. 3, we measured the contact temperature of our detecting module on the foreheads of five human subjects, with the camera running for 35 min with repeated 3-min image acquisition sessions followed by 0.5-min (30 s) breaks in between each session, leading to a duty cycle of 86%. This approach simulated an extreme usage scenario that may occur during hospital scanning. The room temperature was maintained at ∼23°C to mimic typical hospital conditions, where temperatures are usually kept between 20°C and 24°C. The temperature was recorded using a commercial thermocouple (Risepro four channels), positioned between the camera module and the participants’ foreheads. Across recordings from all five subjects, the temperature stabilized after 20 min and never exceeded 41°C, remaining within the comfort temperature range. These results are consistent with those shown in Fig. 2(d) from the silicone hotplate tests with a duty cycle of 100%. Participants were also asked to provide subjective comfort ratings on a scale of “comfortable,” “mild,” “tolerable,” “painful,” or “severe” throughout the 35-min experiment [Fig. 2(d)]. None of the subjects reported intolerable discomfort during the entire duration of the study. Only one participant indicated a comfort level worse than mild, which the subject attributed not to heat but to the pressure of the system due to the tightness of its fasteners. These experiments demonstrate that the multichannel compact SCOS system can operate for extended durations without the risk of heat-induced skin damage while maintaining an adequate level of user comfort.

**Fig. 4:**
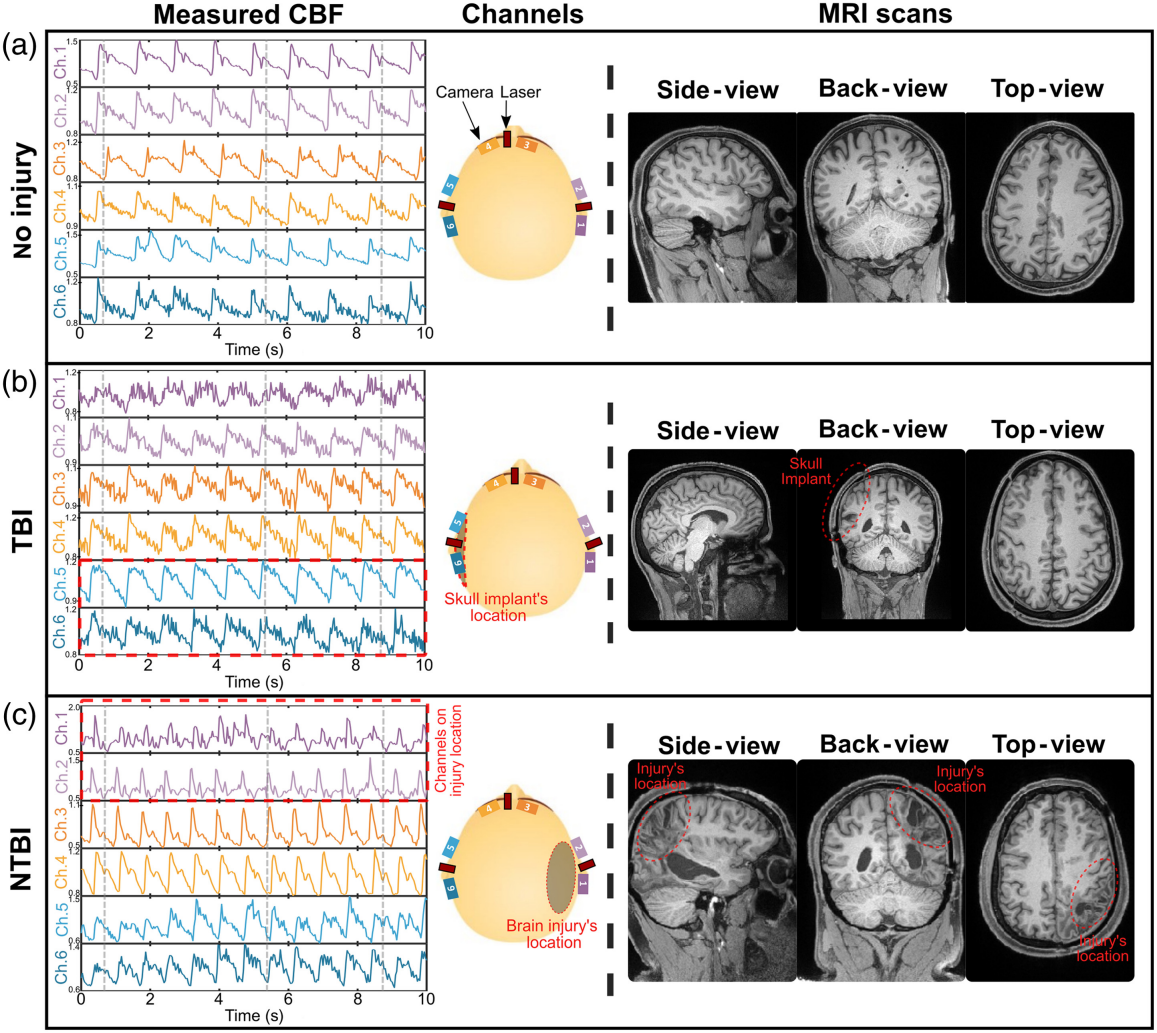
Measured CBF at six different locations and MRI scans of the heads of (a) healthy, (b) TBI, and (c) NTBI subjects. Note that the MRI images are not mirrored; the right side of the image corresponds to the subject’s right side.

Using the six-channel portable system, we measured CBF and CBV on the cohort of seven subjects: five with no history of brain injury (healthy subjects), one with no ongoing major structural brain damage (TBI subject), and one with ongoing brain damage in post-discharge care (NTBI subject). Figure 4 shows the typical CBF measured on a healthy subject [Fig. 4(a)], the TBI subject [Fig. 4(b)], and the NTBI subject [Fig. 4(c)]. As shown, the CBF data for the healthy subject displays synchronized and highly correlated blood flow dynamics across all six channels. A similar pattern is observed in the TBI subject. MRI scans confirmed that neither the healthy subjects nor the TBI subjects had significant brain damage. The CBF data for the NTBI subject exhibited synchronized and correlated blood flow dynamics across all channels except the two channels positioned over the area of brain injury revealed by the MRI scan.

**Fig. 5:**
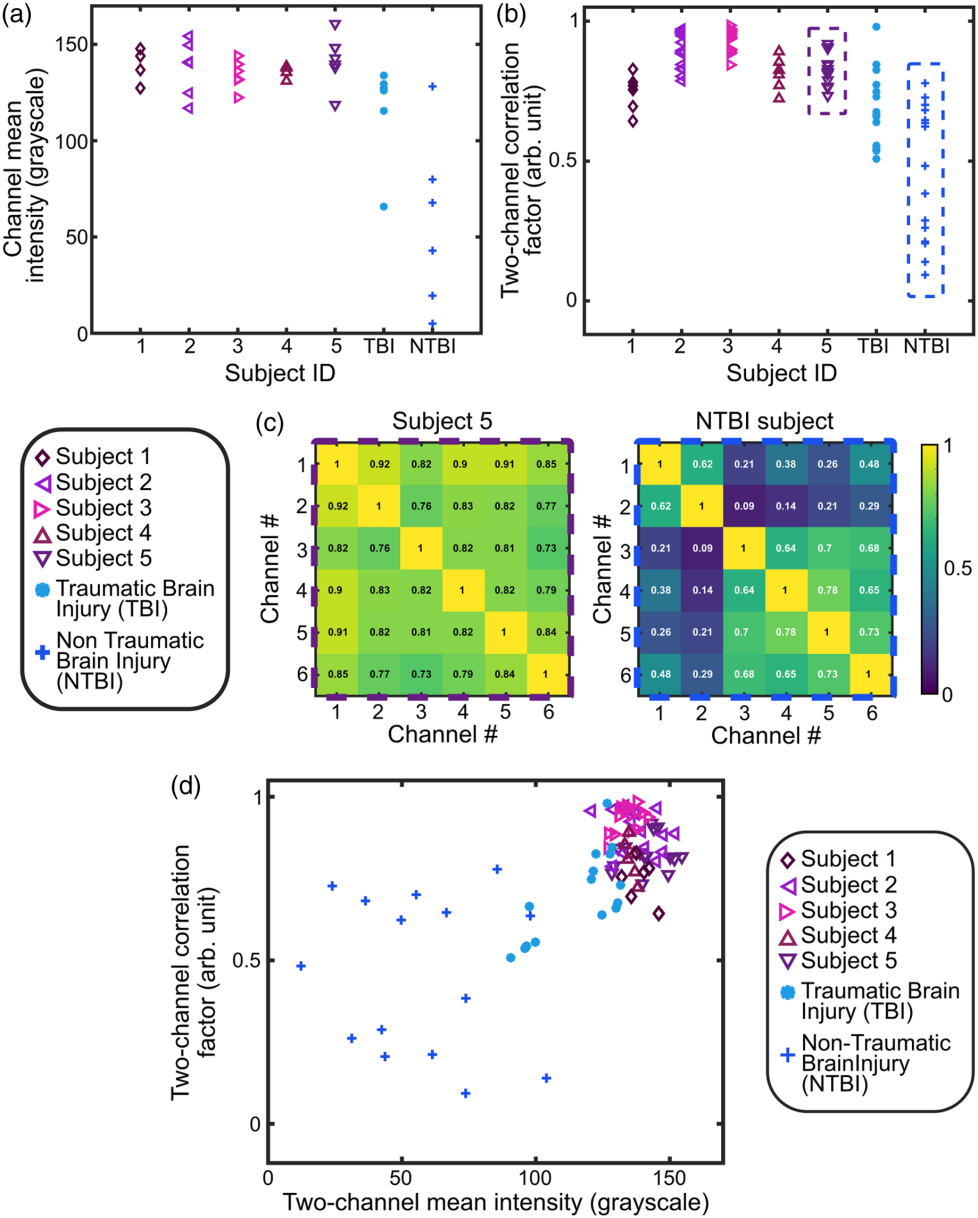
(a) Channel mean intensity and (b) two-channel correlation factor for each of the seven subjects. (c) Correlation matrix of all channels of subject 5 [also shown in Fig. 4(a)] and NTBI subject [also shown in Fig. 4(c)]. (d) Two-channel correlation factor as a function of the two-channel mean intensity for all the subjects.

To further characterize the differences across channels, we calculated the mean intensity of each of the six channels from the CBV recordings [Fig. 5(a)] and the two-channel correlation factor from each subject’s CBF recordings [Fig. 5(b)]. The mean intensity of a channel was calculated as the average spatial intensity of the entire camera image and is expressed in the camera grayscale unit.

The two-channel correlation factor was calculated using the Pearson correlation equation

$$\rho (i,j)=\frac{\sum_{t=1}^{T}(\mathrm{CBFI}(i,t)-\overset{¯}{\mathrm{CBFI}}(i))(\mathrm{CBFI}(j,t)-\overset{¯}{\mathrm{CBFI}}(j))}{\sqrt{\sum_{t=1}^{T}(\mathrm{CBFI}(i,t)-\overset{¯}{\mathrm{CBFI}}(i){)}^{2}\sum_{t=1}^{T}(\mathrm{CBFI}(j,t)-\overset{¯}{\mathrm{CBFI}}(j){)}^{2}}},$$ (7)

where CBFI(i,t) and CBFI(j,t) are the cerebral blood flow index at channels i and j, respectively, $\overset{¯}{\mathrm{CBFI}}(i)$ and $\overset{¯}{\mathrm{CBFI}}(j)$ are the average CBFI over time, and T is the total number of data points in a time trace. Note that the number of possible combinations to calculate the correlation factor is 15 because we correlate two channels together out of six channels (six choose two).

As shown in Figs. 5(a) and 5(b), both the channel mean intensity and two-channel correlation factor were significantly high for all the five healthy subjects. The TBI subject exhibited similar channel mean intensity except for one channel but exhibited a lower correlation factor between channels. The NTBI subject however exhibited significantly lower mean intensity and correlation factor, especially on the four channels located on the back-right side of the head, i.e., the channels closer to the injury location. We note that non-physiological factors such as S-D distance, positioning, coupling, or hair can influence the channel mean intensity measurements of Fig. 5(a). To minimize these, we ensured consistent S-D distances and positioning across all channels and subjects, and all subjects had their head shaved. See the Supplementary Material for a version of Fig. 5 with annotated channels for the TBI and NTBI subjects.

Figure 5(c) presents the correlation matrix between all channels of subject 5, corresponding to the CBF time traces shown in Fig. 4(a), and the NTBI subject, as shown in Fig. 4(c). Although the correlation coefficients between all channels of subject 5 are high (i.e., >0.75), the correlation coefficients between channels of the TBI subject reveal two distinct groups. Channels 1 and 2 are correlated with each other, whereas channels 3 to 6 form a separate correlated group. This suggests that the channels positioned over the injury site exhibit distinct CBF time traces that are different compared with the other channels.

Figure 5(d) shows the two-channel correlation factor [Eq. (7)] as a function of the two-channel mean intensity. The two-channel mean intensity was calculated by averaging the mean intensity of the two channels used to compute the correlation factor. As shown, all subjects except the NTBI subject exhibited high correlation factors and mean intensities, indicating synchronized CBF dynamics with the same pulse waveform across all six channels for the subject. In contrast, the NTBI subject displayed more variability in correlation factors and mean intensities, suggesting regional differences in CBF, likely due to the channels located on the injured region exhibiting different blood dynamics than the other channels.

It is important to note that these observations are preliminary due to the small sample size of only one of each TBI and NTBI subject. A larger study with more brain injury subjects is necessary to draw conclusions about the effectiveness of our multi-channel approach for characterizing traumatic and NTBI. Nonetheless, this demonstrates the potential of the multi-channel SCOS utilizing its high throughput, multi-location, and parallel processing setup to compare signals among channels.

## 4 Discussion

Scaling the single-channel compact SCOS device from our prior work^16^ to a six-channel system presented challenges beyond simply adding more modules. Key hurdles included integrating the system into a lightweight, user-friendly headband, managing high-bandwidth data transfer, ensuring adequate computational power, addressing heat generation from high-speed camera operation, and meeting sanitization standards for clinical use (see the Supplementary Material for more details). Despite these advancements, future work will focus on scaling beyond six channels and improving computational efficiency.

In comparison to diffuse correlation spectroscopy, which typically relies on high-speed photon-counting devices such as single-photon avalanche diode (SPAD) or SPAD arrays operating at >100  kHz sampling rates, along with electronics for synchronization, SCOS offers a more cost-effective alternative by eliminating the need for these specialized components, using a standard camera as the primary sampling device. Our compact SCOS system incorporates specific cost-cutting measures, as presented in Ref. 16. By replacing optical fibers with direct optical coupling and utilizing compact continuous-wave laser diodes and USB board cameras, we have reduced both complexity and production costs. These design choices enhance portability and affordability, making the device suitable for deployment in resource-constrained settings or underserved communities.

In our results shown in Figs. 4 and 5, for the TBI subject, the absence of significant CBF waveform difference across channels compared with the NTBI subject may be attributed to several factors. These include a smaller size and severity of the injury compared with the NBTI subject, a limited number of channels and their placement not being directly over the injury site, and finally the absence of a cerebral perfusion reserve test assessment (e.g., with breath-holding). This highlights the need for a larger cohort of brain injury subjects to validate our findings and better assess the system’s ability to detect subtle or localized abnormalities.

In addition, for the results shown in Fig. 5, one might argue that the low correlation coefficients could be attributed to lower channel mean intensity, i.e., due to a higher noise level in the signal. We ruled out the noise effect as follows: a lower channel mean intensity induces higher noise in CBF waveforms. For two channels with low mean intensities, this would result in a lower CBF two-channel correlation factor because both channels would exhibit higher noise. This would create a linear dependency, where lower mean intensity (i.e., higher noise level) correlates with a lower two-channel correlation factor.

However, the data in Fig. 5(d) do not follow such a linear trend. For the NTBI subject, we observe data points with low two-channel mean intensity (<50) but relatively high two-channel correlation factors (>0.6). This suggests that although the channels located at the injury site [channels 1 and 2 in the NTBI correlation matrix, Fig. 5(c)] exhibit lower mean intensity, they still maintain a high correlation factor of 0.62—comparable to the correlation factors ranging from 0.64 to 0.78 observed for the other four channels not located on the injury site [Fig. 5(c)].

If the altered CBF dynamics at the injury site were solely due to increased noise resulting from low mean intensity, these two channels would not exhibit a high correlation with each other. The observed high correlation factor despite low mean intensity indicates that the altered CBF dynamics at the injury site are not driven by noise but are instead more reflective of physiological differences in CBF dynamics specific to the injury site. Future work will include additional analyses and experiments to further disentangle the contributions of noise versus physiological changes, particularly by increasing the sample size and conducting targeted measurements.

In addition to CBF, we also analyzed CBV time traces at six different locations (see the Supplementary Material). Unlike CBF, CBV dynamics showed no noticeable differences at the site of damage compared with other locations. This underscores the importance of simultaneously measuring both CBF and CBV.

## 5 Conclusion

We demonstrated the effectiveness of the modular compact SCOS systems, expanded to six channels distributed around the head, for monitoring CBF and blood volume at distinct regions of the brain over extended periods. We showed that the device can monitor CBF and CBV for up to 30 min. We plan to extend the recording duration to an hour or more with a 100% duty cycle as contact temperature is not expected to be a limiting factor. The modular design of the system allows flexible positioning of the channels. As such, specific areas of interest can be easily accessed and monitored, with data processed in near real-time on a standard commercial laptop via user-friendly GUI software. Battery-powered laser modules enhance the system’s portability, making it suitable for various environments, including field hospitals, ambulances, examination rooms, or clinics. Aside from the camera cables, the entire system was integrated into a wearable headband that can be secured using zip ties or plastic Velcro straps. Optimized heat dissipation and insulation enable prolonged operation of the system for 30 min or longer while maintaining safe conditions of use and a decent comfort level.

By comparing the six-channel CBF time traces across seven subjects with five healthy subjects, one subject who had mostly recovered from a TBI, and another with a severe NTBI with persistent symptoms, we observed noticeable differences in CBF dynamics at the site of worse structural damage compared with other locations. These findings were corroborated by MRI scans, where the locations of the channels showing altered CBF dynamics matched with the injury site in the MRI scans. The recovered TBI subject exhibited far fewer discrepancies between channels than the NTBI subject with persistent symptoms, and MRI scans showed relatively minor brain injury. A larger study with more brain injury subjects is essential to draw definitive conclusions about the specificity and sensitivity of our multi-channel approach for detecting the cerebrovascular sequelae of brain injury. In future research, we aim to characterize and validate our system in a broader patient population, including both traumatic (TBI) and non-traumatic traumatic brain injuries. By gathering more extensive and quantitative data, we hope to further validate the system’s diagnostic capabilities, which could provide physicians with a valuable and accessible tool for rapid preliminary diagnoses and treatment decisions.

## Supplementary Material



## Data Availability

The data that support the findings of this study are available from the corresponding author upon reasonable request.
