# Supplementary material for: Portable six-channel laser speckle system for simultaneous measurement of cerebral blood flow and volume with potential applications in characterization of brain injury
Source: Neurophotonics. 2025 Jan 24;12(1):015003. doi: 10.1117/1.NPh.12.1.015003 (PMC11758243; doi:10.1117/1.NPh.12.1.015003)
Supplement: Supplementary file 1 [file NPh_012_015003_SD001.pdf]

# Supplementary Material for:
# Portable Six-Channel Laser Speckle System for Simultaneous Measurement of Cerebral Blood Flow and Volume with Potential Applications in Characterization of Brain Injury

**Simon Mahler,[a,†, **] Yu Xi Huang,[a,†] Max Ismagilov,[a] David Álvarez-Chou,[a] Aidin Abedi,[b] J. Michael Tyszka,[c] Yu Tung Lo,[b] Jonathan Russin,[b,d] Richard L. Pantera,[e] Charles Liu,[b,d,*,***] Changhuei Yang[a,***]**
[a] Department of Electrical Engineering, California Institute of Technology, Pasadena, CA 91125, USA
[b]USC Neurorestoration Center and Department of Neurological Surgery, University of Southern California, Los Angeles, CA 90033, USA
[c]Division of Humanities and Social Sciences, California Institute of Technology, Pasadena, CA 91125, USA
[d]Rancho Los Amigos National Rehabilitation Center, Downey, CA 90242, USA
[e]Neurology, Kaweah Health Medical Center, Visalia, CA, USA
[†]These authors contributed equally to this work.
*******These authors co-supervised this work.
***Email:** cliu@usc.edu
****Email:** mahler@caltech.edu, sim.mahler@gmail.com

## Section S1 – Challenges and difficulties encountered in scaling up the SCOS system to six channels

The single-channel compact SCOS device presented in our prior work[16] provided a solid foundation for expansion to a multi-channel SCOS system. However, scaling the system to six channels presented several challenges beyond simply adding more modules, primarily related to headband integration, heat management, sanitization for clinical use, high-bandwidth image data transfer, and ensuring sufficient computational power for real-time data processing.

The first challenge was integrating the six-channel system into a single, user-friendly headband, as shown in Fig. 1. This required designing each component to be compact and lightweight. For the laser source, we used a 9 mm diameter laser diode operating at 808 nm. We custom designed a printed circuit board, connected to a 9V battery, to power supply the laser diode driver (Thorlabs LD1101). Each laser source module weighed approximately 100 g and measured 7 × 6 × 4 cm. The detecting module, shown in Fig. 2(c), weighed approximately 40 g and measured 3.5 × 3.5 × 4 cm. Note that the six-channel system comprises three laser source modules and six detector modules, resulting in a total weight below 550 g, which is comfortably manageable for the human head.

The second challenge arose from switching the camera model from Basler daA1920-160um with IMX392 sensor to Basler daA3840-45um with IMX334 sensor. This quadrupled the number of pixels per frame (from 2.3 to 8.3 million pixels) while halving the sampling rate (from 80 to 40 FPS), effectively doubling the processing load per channel compared to the previous setup[16]. At such high bandwidths, the 5 Gbps USB 3.0 protocol operates near its saturation point. To mitigate this challenge, we capped the sampling rate at 40 FPS, slightly below the 45 FPS maximum supported by the USB 3.0 protocol's bandwidth with an IMX334 sensor. To handle six cameras simultaneously, we required a laptop with six independent USB ports, each connected to a dedicated USB controller to avoid bandwidth conflicts. For this, we selected the Lenovo Legion Pro 5 laptop, which offered both the performance required to handle the processing and 6 USB ports for data transferring. Scaling the system beyond six channels would

require alternative solutions, such as Thunderbolt-to-PCIe docks for laptops or PCIe-to-USB cards for desktop systems.

The decision to use an 8-bit resolution was a trade-off choice between real-time processing needs, data management, and scalability without compromising signal quality. While higher bit-depths, such as 10-bit or 12-bit, provide a higher dynamic range, they increase data size, which in turn demands more bandwidth and computational power, hindering real-time processing, especially in multi-channel systems.

After ensuring real-time image streaming to the PC, the next challenge was processing the data efficiently. Real-time processing was crucial not only for immediate feedback to enhance the user experience but also for compressing the data by saving only output data such as CBF and CBV time traces instead of all the raw camera images. The processing pipeline followed the same structure as in our previous work[16], but the doubling of throughput created a CPU bottleneck. The most computationally demanding task was the pixel-wise division of each image by the average intensity, which changes the data type from int8 to float32. This operation, applied over 8 million pixels per camera across six cameras at 40 fps, posed a significant challenge for CPUs alone. To address this, we integrated GPU-based calculations using CUDA, which significantly alleviated the CPU bottleneck. Transferring tasks such as pixel-wise division, variance, and mean calculations to the GPU improved processing speed and also enhanced power efficiency—critical for portable systems relying on laptops, where the power budget is shared between the CPU and GPU. Using the PyTorch package and entry-level consumer GPUs like the Nvidia GeForce RTX 4070, we achieved real-time processing for the six-channel system. Looking ahead, we anticipate that advances in CPU and GPU technology will further alleviate these limitations. As processors become more powerful and efficient, we expect to eventually be constrained only by CMOS speed limits rather than computational power.

Finally, the camera's high-speed recording generated heat that increases significantly during extended operation. To address this, we incorporated a passive heat management system to dissipate heat effectively to safe levels. For clinical use, all system components were designed with smooth, non-porous surfaces, such as silicone pads, to ensure easy cleaning and sanitization, meeting hygiene standards required for patient-facing applications.

## Section S2 – Choice of the camera for SCOS measurements

The decision to use the Basler daA3840-45um (IMX334) over the daA1920-160um (IMX392) from Ref. [16] was based on several key considerations:

1. **Improved Sensitivity**: The daA3840-45um has a higher quantum efficiency (48% vs. 25% at 808 nm), crucial for our applications with large source-to-detector with low photon count collection.

2. **Smaller Pixel Size**: With a 2 µm pixel pitch compared to the daA1920-160um’s 3.45 µm, the daA3840-45um allows for a reduced camera-to-skin distance (from 6 mm to 5 mm) while maintaining the speckle/pixel length ratio (~0.5).

3. **Cost Efficiency**: The daA3840-45um is slightly more cost-effective than the daA1920-160um.

4. **Camera Shutter**: The daA3840-45um uses a rolling shutter, which could cause slight image smearing when imaging speckles. However, given our system’s speckle-to-pixel length ratio (<1, i.e. multiple speckles per pixel), and our long exposure time of 6 ms that spans multiple decorrelation time periods, the smearing effect from the rolling shutter, occurring a much shorter timescale, is minimal.

5. **Predictable Noise Behavior**: The daA3840-45um exhibits a linear noise response across gain levels, mitigating issues like the 'jumps' observed with the daA1920-160um in low-signal regime, especially in 8-bit mode.

We chose 8-bit (Mono8) over 12-bit (Mono12p) image capturing because 8-bit images are easier to compute and process in int8 instead of int16, and due to USB3.0 bandwidth limitation, we can only record at 30 fps in 12-bit mode while we can record up to 45 FPS in 8-bit mode. Since we calibrate the gain to ensure the grayscale of the images falls in the middle of 0 to 255 range, we can avoid most of the digitization noise. In this case, we prefer using 8-bit images to gain similar sensitivity but with faster frame rates and less demand for processing power. To further support our choice, we conducted tests comparing the two cameras. At each gain setting, 1000 images are captured and the average or the variance of the stack of images is reported.

**Gain Accuracy**: We first investigated the stability of the camera's gain across intensity, which can affect the estimated variance contribution from the shot noise in SCOS measurements. As shown in Fig. S1, across various gain levels, the daA3840-45um mode provided more consistent grayscale value changes, especially in the 8-bit mode at low-light conditions.

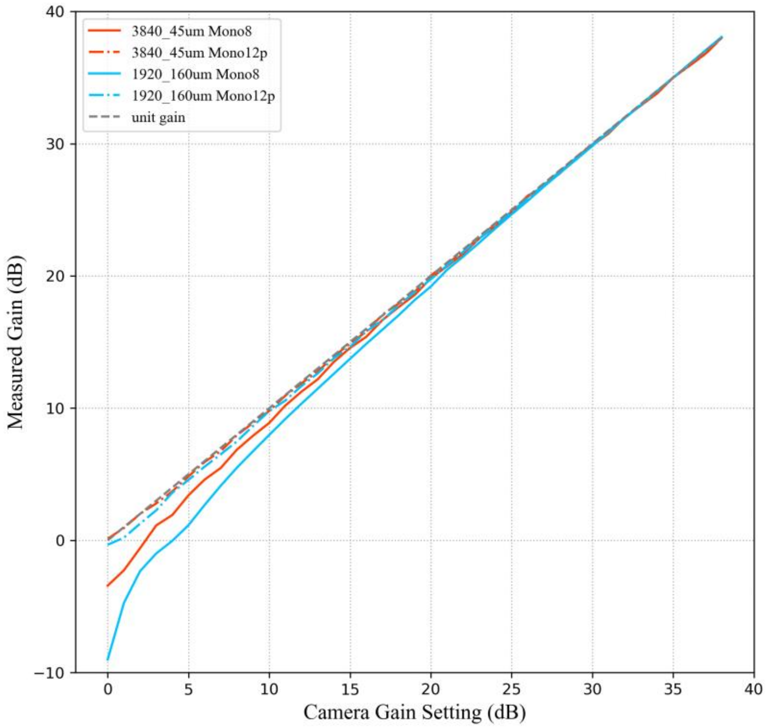


**Fig. S1**: Comparison of camera gain settings versus actual measured gain under constant illumination from a light source that does not saturate the camera sensor, even when at maximum gain level. Deviations between the measured gain and the camera's gain setting are observed at low signal levels due to noise. The daA3840-45um camera adheres more closely to the linear gain curve, demonstrating better stability. Additionally, the daA3840-45um in 8-bit mode performs comparably to its 12-bit mode under moderate to high gain settings.

**Camera Noise**: Noise was measured as the variance of pixel intensity in dark, no-illumination settings, Fig. S2. While the daA1920-160um's larger pixel size provided slightly lower noise, its nonlinear behavior

in 8-bit mode posed challenges for SCOS calibration. In contrast, the daA3840-45um demonstrated linear noise scaling, facilitating reliable calibration.

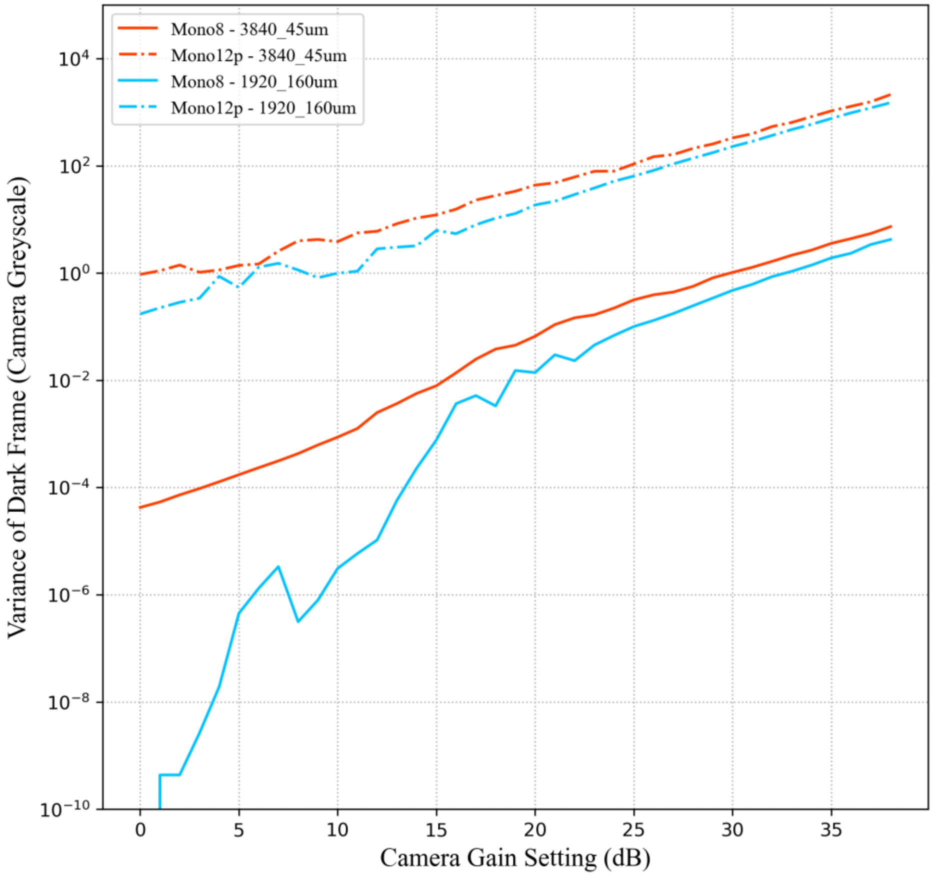


**Fig. S2:** Comparison of camera noise between the daA1920 and daA3840 cameras, measured as the variance of images captured with no illumination at 8-bit (Mono8) and 12-bit (Mono12p) modes. While the daA1920 generally exhibits lower overall noise, it shows instability and unpredictable noise behavior at low camera gain settings, which poses challenges for SCOS implementation. In contrast, the daA3840 demonstrates a mostly linear and stable noise profile, enabling effective calibration and subtraction of the camera's noise contribution to the measured speckle contrast.

**Stability**: In Fig. S3, we calculated the coefficient of variation for mean and variance across the 1,000 images in dark settings at different gain levels. The daA3840-45um consistently exhibited superior stability for intensity and noise, further validating its suitability for SCOS applications.

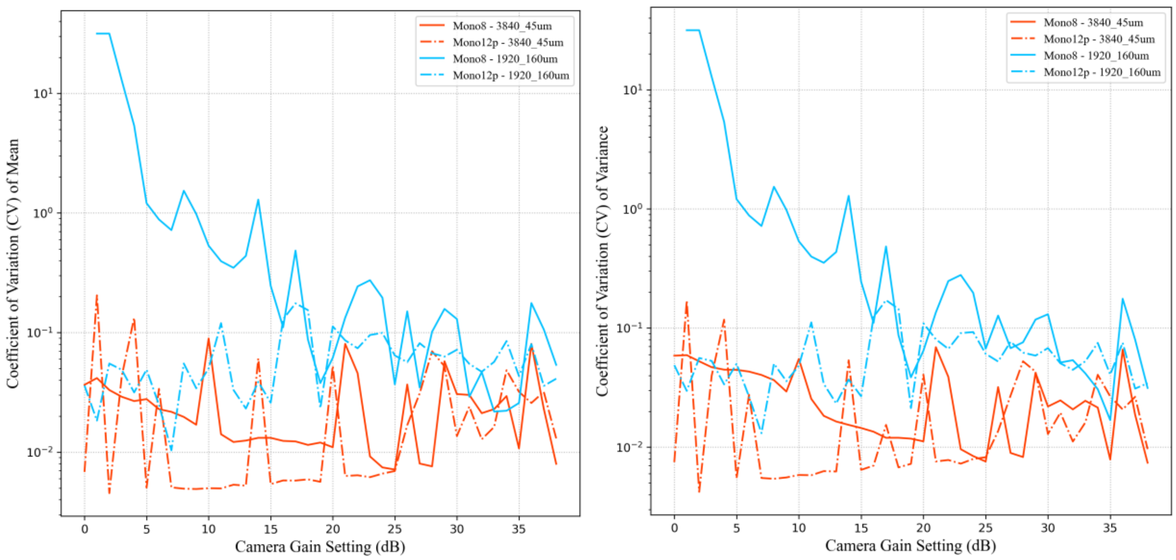


**Fig. S3**: Comparison of the consistency of mean and variance between dark frames for the daA1920 and daA3840 cameras, evaluated using the coefficient of variation (CV) for the means and variances of images captured with no illumination in 8-bit (Mono8) and 12-bit (Mono12p) modes. The daA3840 demonstrated superior consistency, exhibiting lower CV values for both mean and variance across the gain range. Additionally, the 8-bit mode of the daA1920 showed lower CV than its 12-bit mode. This improved consistency enhances processing accuracy when accounting for dark noise and read noise in frame-to-frame analysis.

While the Basler daA1920-160um board camera was effective in our previous work of Ref. [16], the Basler daA3840-45um board camera offers notable improvements in sensitivity, noise predictability, and stability. These enhancements contribute to more consistent SCOS calibration and analysis, particularly in low-light conditions.

## Section S3 – Performance comparisons of the batteries

Figure 2(a) of the main text showed the laser output power for three different power sources: a 9V DC bench power supply, an alkaline battery [Amazon Basics 6LR61], and a rechargeable lithium-ion battery [EBL® 6F22]. We also tested a zinc chloride battery [Thunderbolt Magnum9V], but it lasted less than 5 minutes, making it unsuitable for use in the system. As shown in Fig. 2, the power sources provided the laser with an optical power of approximately 66 mW over time. The DC power supply maintained a constant output, while the alkaline and rechargeable lithium-ion batteries' power decreased to zero after a few hours due to discharge.

We repeated those measurements over five different realizations and measured the duration of lasing. On average, the alkaline battery lasted an average of 3.2 ± 0.2 hours, and the rechargeable lithium-ion battery lasted 3.7 ± 0.2 hours. Larger instabilities occurred during the first 5 to 10 minutes where the laser power increased by about 3 mW due to the printed circuit board’s onset time (specifically, the charging/discharging of capacitors), the laser diode’s lasing dynamics, and the initial quick discharge of the battery’s voltage. After the onset period, the laser's optical power remained stable at around 66 mW, with fluctuations of less than 0.6 mW for the alkaline battery, 0.4 mW for the rechargeable lithium-ion battery, and 0.3 mW for the DC power supply. We estimated that a laser power fluctuation of 0.5 mW or less during a recording is needed for stable measurements of CBF and CBV. Therefore, the system should be warmed up for 5 to 10 minutes before recording begins. To facilitate this, we have added a switch plug

in front of the laser diode mount to allow the system to warm up while not exposing the subject with laser light.

We also measured the voltage of the power supply with an oscilloscope, Fig. 2(b), and observed that the battery discharge curves in Fig. 2(b) closely match the optical power curves in Fig. 2(a). The DC power curve remained constant without fluctuation, even in the first few minutes. Compared to the rechargeable lithium-ion battery, the alkaline battery had a larger discharge over the first 3.5 hours, explaining the higher stability of optical power with the rechargeable battery. The rechargeable lithium-ion batteries weighed on average 25 g, about 50% lighter than the alkaline batteries, which weighed on average 48 g. Due to their superior lasing stability, longer lasing period, and lighter weight, rechargeable lithium-ion batteries are more suitable for our compact SCOS system.

As shown in Figs. 2(a) and 2(b), there is a decrease in laser power that aligns with the battery's discharge curve, potentially causing variations in blood dynamics measurements. We identified three regimes where this may occur:

1) **Initial Power-On Instabilities:** During the first 5–10 minutes of operation, large increases in optical power (see inset of Fig. 2(a)) occur due to a combination of battery discharge initialization and "warming-up" of electrical components (e.g., capacitors). These fluctuations can cause significant CBV variations if measurements are taken immediately after powering on the system. To mitigate this, we recommend a warming period of 5–10 minutes before starting data acquisition.
2) **Depletion Phase:** The battery depletion typically occurred after 3.5 hours of laser operation. For alkaline batteries, this process is gradual, leading to extended periods of CBV instability. In contrast, rechargeable lithium-ion batteries experience a more abrupt depletion phase (within ~3 minutes) due to an internal voltage cutoff mechanism. Given the shorter and more predictable nature of their depletion phase, we recommend using rechargeable lithium-ion batteries to minimize CBV instabilities.
3) **Gradual Discharge Phase:** Between the initial and depletion phases, the battery voltage decreases gradually over 3+ hours, leading to a slight reduction in laser power (a few milliwatts), Figs. 2(a) and 2(b). This can cause a slow upward trend in CBV measurements, particularly during extended acquisitions. However, such variations primarily affect the DC baseline of the CBV signal and can be digitally filtered by using a baseline window of 1 minute. While this workaround is effective, we agree that a more robust solution is needed. Future system iterations will focus on adding feedback control loops or advanced power regulation mechanisms to stabilize optical power throughout the battery's discharge cycle.

## Section S4 – Figure 5 of main text with annotated channels for the TBI and NTBI subjects

In this section, we show Fig. 5 of main text with but with annotated channels for the TBI and NTBI subjects, see Fig. S4.

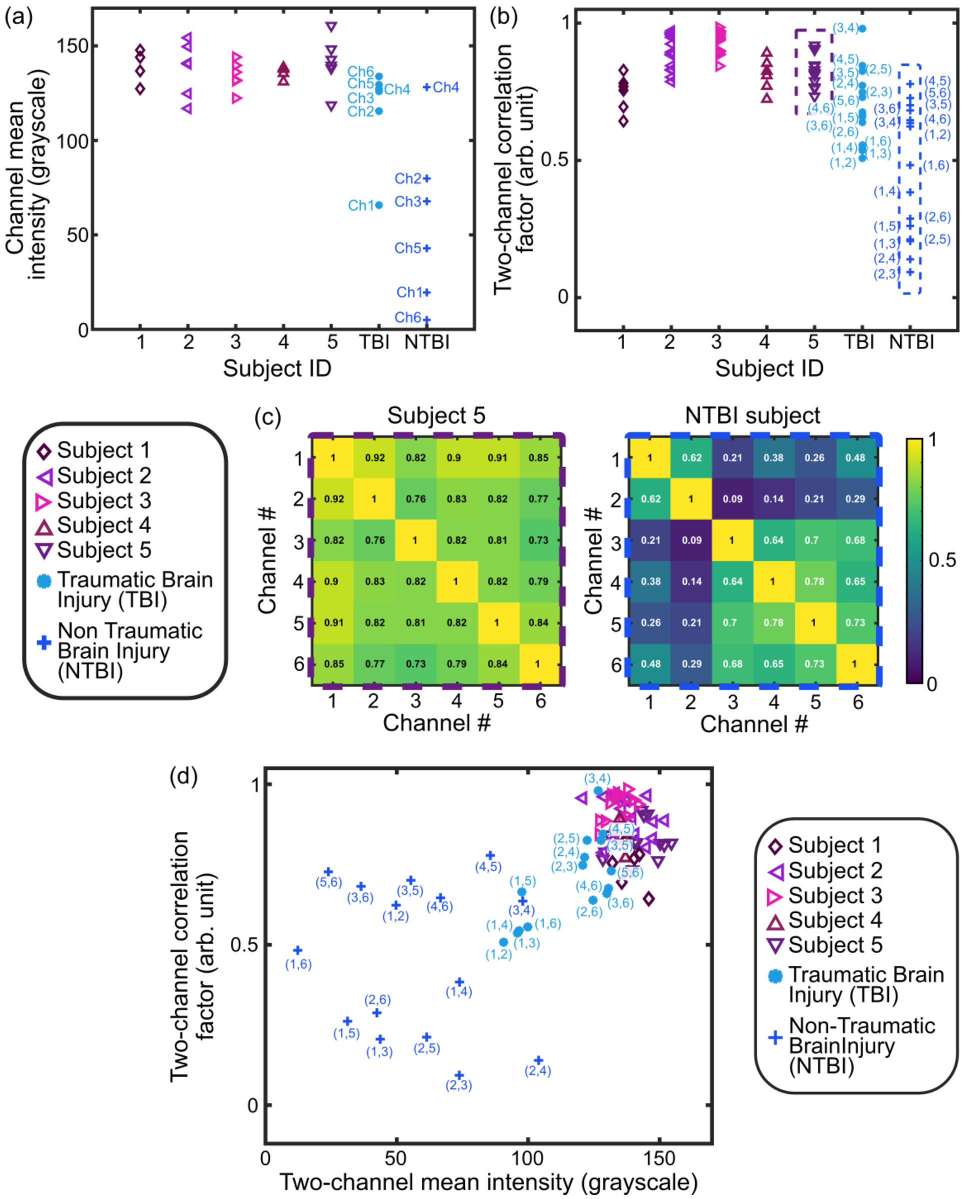


**Fig. S4:** Figure 5 of main text with annotated channels for the TBI and NTBI subjects.

## Section S5 – CBF time trace over a 20-minute period

In this section, we show the CBF over a 20-minute period recorded on a subject who remained still throughout the recording (Subject 3 of Fig. 3). The device operated with an 86% duty cycle, featuring 180-second imaging sessions followed by 30-second breaks, as in Figure 3. Figure S5(a) displays the complete CBF signal over approximately 1,250 seconds, showing stable CBF index (CBFI) values throughout the recording. Figure S5(b) focuses on the final recording cycle, revealing a consistent and repetitive CBF signal. Further zoomed views in Figures S5(c) and S5(d) illustrate clear cardiac cycles, similar to those in Figure 1. These findings indicate that the SCOS system maintains signal integrity during extended operation, with minimal impact from laser noise fluctuations or battery voltage drift. Note that

to obtain the results of Fig. S5, it is crucial that the subject head remains still during the entire recording, as even slight head movements can introduce sharp spikes in the CBF signal, as in Figure S5(a).

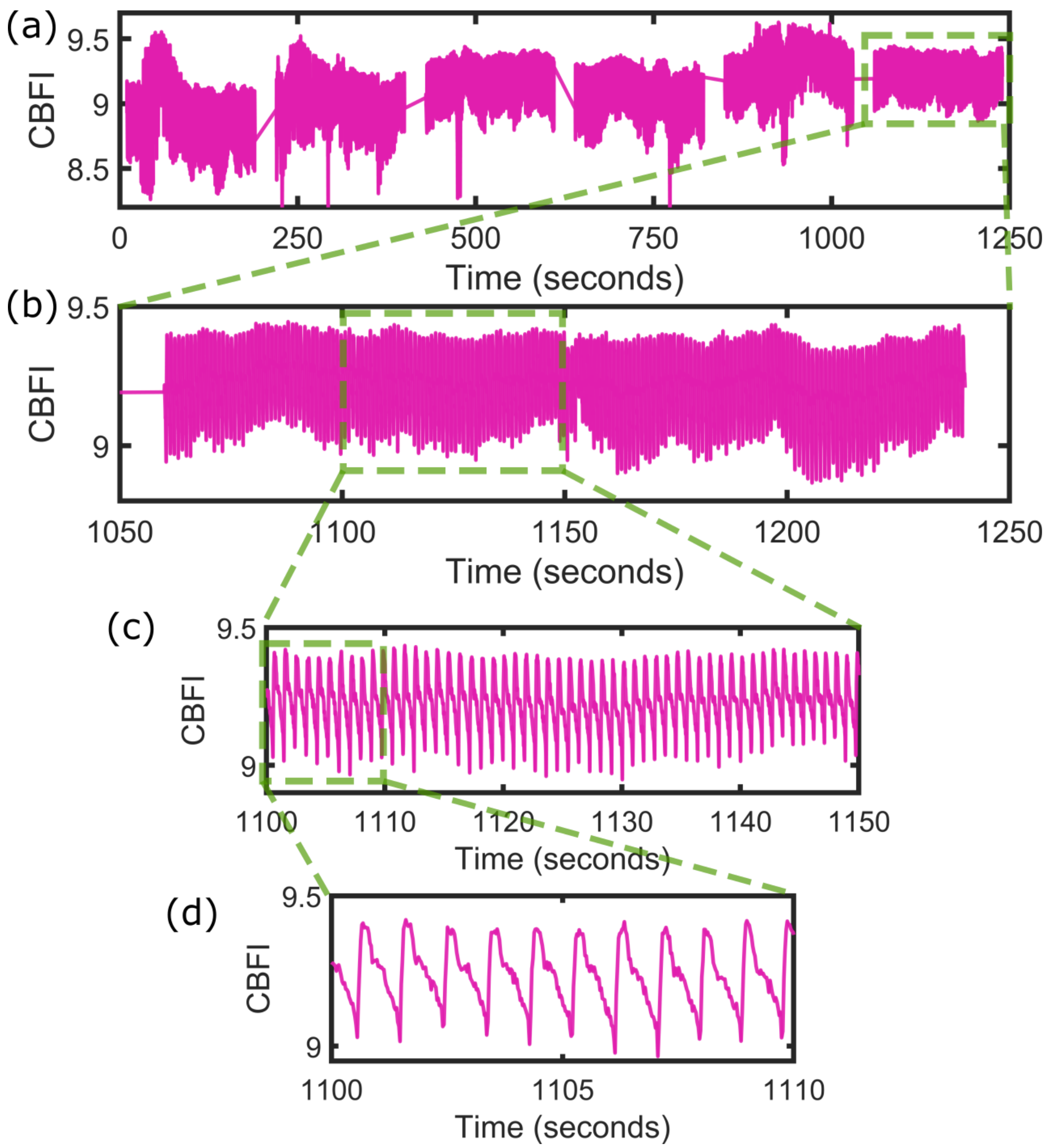


**Fig. S5:** Monitoring of cerebral blood flow (CBF) over an extended period of 20-minute. (a) The complete CBF signal, demonstrating CBFI values throughout the recording. (b) Zoom-in on the last recording cycle. (c) A 50-second zoomed view of the CBF signal. (d) A 10-second zoomed view.

## Section S6 – Six-channel cerebral blood volume (CBV) results

In this section, we present Fig. S6, which is a modified version of Fig. 4 from the main text, but with CBV time traces instead of CBF. As shown in Figure S6, there are no noticeable differences in CBV dynamics at the site of damage compared to other locations. This highlights the importance of simultaneously measuring both CBF and CBV, as CBF may contain distinct physiological information not captured by CBV.

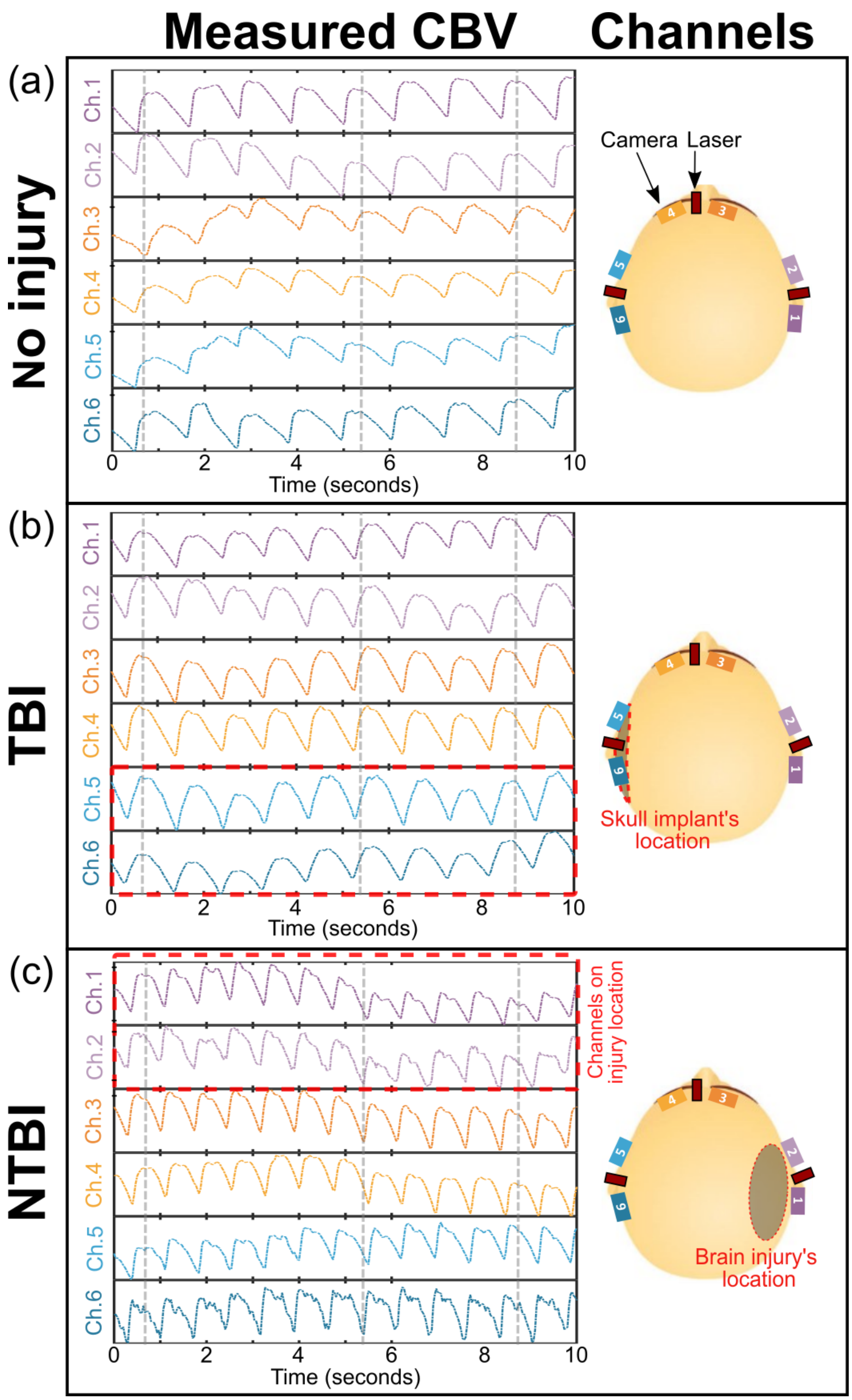


**Fig. S6:** Measured cerebral blood volume (CBV) at six different locations of the heads of (a) healthy, (b) TBI, and (c) NTBI subjects.

References (numbered as they appear in the main manuscript):

16.Y. X. Huang et al., "Compact and cost-effective laser-powered speckle contrast optical spectroscopy fiber-free device for measuring cerebral blood flow," J. Biomed. Opt. 29(06), 067001 (2024) [doi:10.1117/1.JBO.29.6.067001].

17.C. G. Favilla et al., “Validation of the Openwater wearable optical system: cerebral hemodynamic monitoring during a breath-hold maneuver,” Neurophoton. 11(01) (2024) [doi:10.1117/1.NPh.11.1.015008].
18.B. Kim et al., “Measuring human cerebral blood flow and brain function with fiber-based speckle contrast optical spectroscopy system,” Commun Biol 6(1), 844 (2023) [doi:10.1038/s42003-023-05211-4].
19.T. Dragojević et al., “Compact, multi-exposure speckle contrast optical spectroscopy (SCOS) device for measuring deep tissue blood flow,” Biomed. Opt. Express 9(1), 322 (2018) [doi:10.1364/BOE.9.000322].
